# A shift towards early-age desexing of cats under veterinary care in Australia

**DOI:** 10.1038/s41598-020-79513-6

**Published:** 2021-01-18

**Authors:** Loic Mazeau, Claire Wylie, Lara Boland, Julia A. Beatty

**Affiliations:** 1 Faculty of Science, Sydney School of Veterinary Science, University of Sydney, Sydney, NSW 2006 Australia; 2 Ecole Nationale Vétérinaire de Toulouse, 31300 Toulouse, France; 3 Department of Veterinary Clinical Sciences & Centre for Companion Animal Health, Jockey Club College of Veterinary Medicine and Life Sciences, City University, Hong Kong, SAR China

**Keywords:** Ecology, Zoology

## Abstract

The global problem of unowned domestic cats, driven by their phenomenal reproductive success, carries significant economic, animal welfare and biodiversity costs. Desexing owned cats prior to puberty prevents unwanted litters that contribute to unowned cat populations. The prevalence and predictors of desexing, and the age at which surgery was carried out were investigated using anonymized electronic patient records in the VetCompass Australia database of cats presented to veterinary practices. Of 52,941 cats born between 2010 and 2017, 83.6% were desexed. Among 7463 desexed females, 21.5% had been desexed by 4 months of age, 59.8% by 6 months and 85.4% by 1 year. Sex, breed, location and socioeconomic indices significantly influenced desexing status and age at surgery. Cats born between 2010 and 2017 had greater odds of being desexed than cats born between 1995and 2009 at each age cut-off (≤ 4 months [OR 1.76, CI_95_ 1.58–1.97], ≤ 6 months [OR 1.50, CI_95_ 1.38–1.62] and ≤ 1 year [OR 2.33, CI_95_ 2.11–2.57] *p* < 0.001). Most cats presented to veterinarians in Australia are desexed. Compared with cats born before 2010, cats born later are significantly younger at desexing but, even so, many cats would have reached sexual maturity before surgery. These findings will inform the design of front-line strategies promoting prepubertal desexing and they demonstrate, for the first time, a shift towards desexing younger cats.

## Introduction

A proactive approach to curb the breeding of owned cats is essential to prevent unwanted litters. Females can produce 1 to 3 litters, of 1 to 6 kittens, every year^1^. The early onset of puberty, usually at between 5 to 9 months of age^1–3^, but sometimes as young as 3.5 months of age in females^4^, often comes as a surprise to owners^5^. Unwanted litters are both common^5,6^, and a frequent reason for relinquishment to shelters^4,7,8^, where cats under 6 months of age consistently comprise the majority of cats surrendered^9–12^. Unwanted kittens contribute to stray cat populations^13^, raising concerns for their welfare, and exerting additional stress on some sympatric wildlife populations already impacted by habitat loss, global warming and other predators^14,15^.

Surgical gonadectomy, known as desexing or neutering, is a routine procedure that effectively prevents unwanted litters if carried out prior to puberty. Early-age desexing (EAD), or prepubertal desexing, is performed when kittens reach 1 kg body weight, which is usually by 4 months old. In contrast, the traditional age for desexing is 6 months^16,17^. EAD has been practiced routinely at animal shelters in the USA for over 30 years^18^, and subsequently embraced more widely organizations including by the International Society of Feline Medicine^19^, the Royal Society for Prevention of Cruelty to Animals in Australia^20^ and The Cat Group, UK^21^. Concerns that EAD might increase anaesthetic risk or cause orthopaedic, behavioural and lower urinary tract diseases, have now been addressed by an evidence-base that supports that EAD is not only safe, but offers advantages over traditional age desexing, including more rapid recovery times and earlier socialization^4,17,22–30^.

The impact of EAD on overpopulation will be realized only when it is widely adopted in veterinary practice. However, broad support from the veterinary profession for EAD has not yet been achieved. Most veterinarians surveyed in Australia in 2013 recommended desexing of cats at 6 months of age, and perceived anaesthetic risk as the major reason to not recommend EAD^31^. In 2015, EAD was advocated by only one in three teaching staff at Australian veterinary schools, and most veterinary students graduated without training in EAD^32,33^. In the Australian Capital Territory (ACT), where desexing by 3 months has been mandated since 2007, only 5/52 veterinarians surveyed made this recommendation to their clients^34^. Overall, these studies suggest that the attitudes and recommendations of Australian veterinarians regarding the age of desexing for cats are inconsistent with current evidence and legislation.

Identifying variables that have a discernible effect on both whether a cat is desexed and, crucial to population control, the age at which surgery is carried out, can assist in directing resources to promote EAD. To date, studies investigating age at desexing are limited. A survey of cat owners in the UK found that 92% of all cats older than 6 months, but only 66% percent of cats aged 6–12 months, were desexed^6^. Among 900 cats presented for microchipping in Western Australia (WA), only 49% and 28% of cats under 2 years of age had been desexed in 2012 and 2013 respectively, compared with over 90% of cats aged 2 years or more^35^.

The availability of the 2017 VetCompass Australia (VCA) database provided an opportunity to apply large-scale analysis of anonymized electronic patient records (EPR), for the first time, to the investigation of desexing practices in cats presented to first-opinion veterinary practices across Australia^36^. VCA collates EPRs from veterinary practices into a database for epidemiological studies. Modelled on the VetCompass Animal Surveillance project developed by the Royal Veterinary College, UK^37^, VCA is the largest companion animal clinical data repository in Australia. This study had three aims (1) to investigate the prevalence and predictors of desexing among cats presented to first-opinion practices in Australia, (2) to investigate the age at which desexing was performed and its predictors and (3) to compare age at surgery in cats born between 2010 and 2017 with that in cats born between 1995 and 2009, to investigate changes in practice.

## Results

### Prevalence and predictors of desexing

Desexing status was recorded for 52,941 cats born between 2010 and 2017 inclusive of which 83.6% were desexed (CI_95_ 83.3–83.9%). All variables had a significant influence on desexing status in the univariable logistic regression. Variables that remained significant in the multivariable logistic regression are presented in Table 1. The odds of being desexed were significantly higher in males and mixed breed cats compared with females and purebred cats, respectively. Among different States, cats from SA and VIC had higher odds of being desexed compared with cats in NSW, whereas the odds were lower in cats from QLD. Socio-economic conditions influenced desexing status; being desexed was less likely in areas with less access to resources (Remoteness area (RA), more low income households (Index of Economic Resources (IER)) and greater relative socioeconomic disadvantage (Index of Relative Socio-Economic Disadvantage (IRSD) (Table 1). The proportion of desexed cats correlated strongly with the IER (y = 0.0137x + 0.8294, R^2^ = 0.951) and the IRSD (y = 0.0168x + 0.8137, R^2^ = 0.9756) in linear regression analyses.

**Table 1:** Prevalence and predictors of being desexed in an Australian cat cohort born between 2010 to 2017 inclusive.

Variable	Category	Number of cats	Prevalence (%) of desexed cats and CI_95_	Odds Ratio	*p*-value
Total population	–	52,941	83.6 (83.3–83.9)	–	–
Sex	Female	26 348	81.0 (80.5–81.4)	*Reference*	
	Male	26 279	87.0 (86.6–87.5)	1.37 (1.29–1.45)	** < 0.001**
Breed	Mixed breed	41 702	84.8 (84.5–85.2)	*Reference*	
	Purebred	10 411	83.4 (82.7–84.1)	0.79 (0.70–0.88)	** < 0.001**
State	NSW	13 555	80.3 (79.6–80.9)	*Reference*	
	ACT	1 059	88.2 (86.3–90.2)	1.03 (0.82–1.30)	0.77
	QLD	19 502	84.2 (83.7–84.7)	0.73 (0.68–0.78)	** < 0.001**
	SA	2 009	87.9 (86.5–89.3)	1.47 (1.24–1.75)	** < 0.001**
	VIC	11 364	86.8 (86.2–87.4)	1.28 (1.17–1.39)	** < 0.001**
	WA	1 109	85.3 (83.2–87.3)	0.96 (0.77–1.19)	0.70
Remoteness area	MCA	39 252	84.4 (83.7–85.2)	*Reference*	
	Other	9 285	82.1 (81.7–82.4)	0.87 (0.80–0.94)	** < 0.001**
Index of economic resources (quantile)	1	9 094	81.2 (80.4–82.0)	*Reference*	
	2	8 876	82.3 (81.5–83.0)	1.05 (0.95–1.15)	0.27
	3	10 990	83.5 (82.8–84.1)	1.06 (0.96–1.17)	0.23
	4	10 679	86.3 (85.6–86.9)	1.17 (1.05–1.31)	**0.003**
	5	8 883	86.5 (85.8–87.2)	1.19 (1.05–1.35)	**0.004**
Index of relative socio-economic disadvantage (quantile)	1	4 335	79.5 (78.3–80.7)	*Reference*	
	2	6 466	80.5 (79.5–81.4)	1.14 (1.02–1.27)	**0.01**
	3	14 844	83.7 (83.1–84.3)	1.34 (1.20–1.51)	** < 0.001**
	4	10 635	85.3 (84.6–86.0)	1.31 (1.16–1.49)	** < 0.001**
	5	12 242	86.6 (86.0–87.2)	1.52 (1.32–1.74)	** < 0.001**

Significant values (*p* < 0.05) are indicated in bold.

*CI* confidence interval, *MCA* major cities of Australia.

### Age at desexing and its predictors

Age at desexing was identified for 16,085 cats born between 2010 and 2017 inclusive. Sex and breed, recorded for ≥ 99% of the population, showed 53.9% males, and a majority of mixed breed cats (80.6%) (Table 2). Of this desexed cat population, 23.2% had been desexed by 4 months of age and 62.6% by 6 months of age. The median age at which cats were desexed was 6 months old (range < 1 to 93 months). All variables studied had a significant influence on the age at desexing in the univariable logistic regression. The results of the multivariable logistic regression are shown in Table 3. The odds of desexing ≤ 4 months were significantly influenced by all variables except Remoteness Area. In pairwise comparisons, EAD was more likely for males. A significant interaction between sex and breed was identified, with purebred males being more likely to be desexed than purebred females at ≤ 4 months (iOR 1.89 (CI_95_1.49–2.43, *p* < 0.001) and at ≤ 6 months (iOR 1.37 (CI_95_1.15–1.63, *p* < 0.001). The odds of desexing at ≤ 4 months were 2.6 times higher in mixed breed cats compared with purebreds, whereas no difference was seen at 6 months.

**Table 2:** Prevalence (%) of cats desexed before each age cut-off in an Australian cohort of desexed cats born between 2010 and 2017 inclusive.

Variable	Category	Number of cats	Age at desexing (CI_95_)			
			$\le$ 4 months	$\le$ 6 months	$\le$ 1 year	$\le$ 2 years
Sex	Female	7377	21.5 (20.6–22.5)	59.8 (58.6–60.9)	85.4 (84.6–86.2)	94.5 (94.0–95.0)
	Male	8696	24.5 (23.6–25.4)	65.2 (64.2–66.2)	88.5 (87.9–89.2)	95.4 (95.0–95.8)
Breed	Domestic	12,977	25.0 (24.3–25.7)	62.4 (61.6–63.3)	86.8 (86.2–87.3)	95.0 (94.6–95.4)
	Pure breed	3069	15.3 (14.0–16.6)	64.1 (62.4–65.8)	89.2 (88.1–90.3)	95.6 (94.8–96.3)
Season of birth	Autumn	3203	25.6 (24.0–27.1)	64.3 (62.7–66.0)	86.2 (85.1–87.4)	95.5 (94.8–96.2)
	Spring	6115	25.6 (24.5–26.7)	66.6 (65.4–67.8)	91.3 (90.5–92.0)	97.4 (97.0–97.8)
	Summer	4663	22.6 (21.4–23.8)	64.0 (62.7–65.4)	88.5 (87.6–89.4)	96.2 (95.6–96.7)
	Winter	2104	13.8 (12.3–15.3)	50.6 (48.4–52.7)	79.8 (78.1–81.6)	91.8 (90.6–92.9)
State	NSW	4655	22.9 (21.7–24.1)	61.3 (59.9–62.6)	85.4 (84.4–86.4)	93.8 (93.1–94.5)
	ACT	285	18.5 (14.0–23.1)	63.0 (57.4–68.6)	88.1 (84.4–91.8)	95.4 (93.0–97.8)
	QLD	5833	16.4 (15.5–17.4)	60.1 (58.8–61.3)	87.6 (86.8–88.4)	95.5 (95.0–96.1)
	SA	594	12.9 (10.2–15.6)	60.2 (56.3–64.1)	86.7 (84.0–89.4)	94.8 (93.1–96.6)
	VIC	3589	34.2 (32.6–35.7)	67.4 (65.9–69.0)	87.9 (86.8–88.9)	95.3 (94.7–96.0)
	WA	154	33.1 (25.6–40.5)	68.1 (60.8–75.5)	92.8 (88.7–96.9)	98.7 (96.9–100)
Remoteness area	MCA	11,500	21.8 (21.1–22.6)	62.9 (62.0–63.7)	87.6 (87.0–88.2)	95.3 (94.9–95.6)
	Other	3596	25.3 (23.9–26.8)	60.7 (59.1–62.2)	85.3 (84.1–86.4)	94.0 (93.3–94.8)
Index of economic resources (quantile)	1	2862	19.1 (17.7–20.5)	58.7 (56.9–60.5)	84.8 (83.5–86.1)	93.9 (93.0–94.8)
	2	3147	22.0 (20.6–23.5)	61.4 (59.7–63.1)	87.1 (85.9–88.3)	95.1 (94.4–95.9)
	3	3794	25.7 (24.3–27.1)	60.4 (58.9–62.0)	85.0 (83.9–86.2)	93.8 (93.0–94.5)
	4	3009	22.0 (20.5–23.5)	64.2 (62.4–65.9)	88.8 (87.7–90.0)	96.1 (95.4–96.7)
	5	2281	23.8 (22.1–25.6)	68.9 (67.0–70.8)	90.8 (89.6–91.9)	96.6 (95.9–97.4)
Index of relative socio-economic disadvantage (quantile)	1	1536	22.7 (20.6–24.8)	57.3 (54.8–59.8)	84.2 (82.4–86.0)	93.8 (92.6–95.0)
	2	2231	19.7 (18.1–21.4)	59.7 (57.6–61.7)	85.1 (83.6–86.5)	93.7 (92.7–94.7)
	3	5443	24.8 (23.7–26.0)	60.2 (58.9–61.5)	85.5 (84.6–86.5)	94.4 (93.8–95.0)
	4	2919	20.3 (18.9–21.8)	63.2 (61.4–64.9)	88.3 (87.1–89.4)	95.6 (94.9–96.3)
	5	2964	23.2 (21.7–24.8)	70.1 (68.5–71.8)	91.6 (90.6–92.6)	96.9 (96.3–97.5)

*CI* confidence interval, *MCA* major cities of Australia.

**Table 3:** Variables significantly associated with age at desexing using a multivariable logistic regression model in an Australia cat cohort born 2010–2017 inclusive.

Variable	Category	Age at desexing (CI_95_)							
		$\le 4$ months		$\le$ 6 months		$\le 1$ year		$\le 2$ years	
		OR	p-value	OR	p-value	OR	p-value	OR	p-value
Sex	Female	*Reference*		*Reference*		*Reference*		*Reference*	
	Male	1.18 (1.09–1.29)	** < 0.001**	0.82 (0.66–1.03)	0.09	1.18 (1.06–1.31)	**0.003**	0.95 (0.79–1.14)	0.64
Breed	Domestic	*Reference*		*Reference*		*Reference*		*Reference*	
	Pure breed	0.39 (0.30–0.50)	** < 0.001**	0.93 (0.75–1.17)	0.57	1.01 (0.74–1.40)	0.93	0.64 (0.49–0.84)	**0.001**
Season of birth	Autumn	*Reference*		*Reference*		*Reference*		*Reference*	
	Spring	0.95 (0.79–1.15)	0.64	1.20 (1.06–1.34)	**0.002**	1.68 (1.44–1.95)	** < 0.001**	1.76 (1.39–2.24)	** < 0.001**
	Summer	0.81 (0.66–1.00)	0.05	0.96 (0.85–1.09)	0.58	1.25 (1.07–1.45)	**0.005**	1.19 (0.94–1.51)	0.13
	Winter	0.57 (0.43–0.75)	** < 0.001**	0.60 (0.52–0.70)	** < 0.001**	0.57 (0.48–0.67)	** < 0.001**	0.51 (0.40–0.64)	** < 0.001**
State	NSW	*Reference*		*Reference*		*Reference*		*Reference*	
	ACT	0.44 (0.17–0.97)	0.06	0.86 (0.67–1.12)	0.28	0.91 (0.62–1.37)	0.63	1.01 (0.55–2.03)	0.96
	QLD	0.67 (0.53–0.84)	** < 0.001**	0.94 (0.86–1.02)	0.18	1.19 (1.05–1.34)	**0.005**	1.39 (1.15–1.69)	** < 0.001**
	SA	0.45 (0.24–0.80)	**0.009**	0.96 (0.80–1.15)	0.70	1.18 (0.91–1.56)	0.21	1.49 (0.96–2.43)	0.08
	VIC	1.45 (1.14–1.84)	**0.002**	1.20 (1.09–1.33)	** < 0.001**	1.13 (0.98–1.31)	0.08	1.39 (1.10–1.77)	**0.005**
	WA	0.36 (0.08–1.20)	0.12	1.10 (0.78–1.58)	0.56	1.57 (0.88–3.13)	0.15	3.18 (0.99–19.4)	0.10
Remoteness area	MCA	*Reference*		*Reference*		–	–	–	–
	Other	0.93 (0.69–1.24)	0.63	1.21 (0.98–1.49)	0.06	–	–	–	–
Index of economic resources (quantile)	1	*Reference*		–	–	–	–	–	–
	2	1.38 (1.17–1.62)	** < 0.001**	–	–	–	–	–	–
	3	2.02 (1.69–2.41)	** < 0.001**	–	–	–	–	–	–
	4	1.60 (1.31–1.94)	** < 0.001**	–	–	–	–	–	–
	5	1.77 (1.42–2.20)	** < 0.001**	–	–	–	–	–	–
Index of relative socio-economic disadvantage (quantile)	1	*Reference*		*Reference*		*Reference*		*Reference*	
	2	0.86 (0.71–1.04)	0.12	1.02 (0.83–1.25)	0.82	1.22 (1.01–1.48)	**0.03**	1.36 (1.00–1.85)	**0.04**
	3	0.80 (0.66–0.98)	**0.03**	0.93 (0.78–1.11)	0.45	1.18 (1.00–1.39)	**0.04**	1.27 (0.97–1.64)	0.06
	4	0.68 (0.54–0.85)	** < 0.001**	1.04 (0.85–1.27)	0.64	1.44 (1.19–1.74)	** < 0.001**	1.55 (1.15–2.09)	**0.004**
	5	0.76 (0.60–0.96)	**0.02**	1.35 (1.11–1.65)	**0.002**	2.06 (1.68–2.53)	** < 0.001**	2.24 (1.62–3.11)	** < 0.001**

Significant values (*p* < 0.05) are indicated in bold.

*CI* confidence interval, *MCA* major cities of Australia.

Cats born in winter had the lowest odds of being desexed before either 4 or 6 months of age. Between states, the odds of EAD were highest for cats from Victoria, followed by NSW, QLD, ACT, and SA, and these differences between states were significant or borderline (ACT *p* = 0.05). Socioeconomic indices RA, IED and IRSD significantly influenced the chances of EAD but in different directions; EAD was least likely in areas with the lowest annual income, but most likely in areas of greatest socioeconomic disadvantage.

### Comparison of age at desexing surgery in consecutive Australian cat cohorts

#### Descriptive results for cohort born 1995–2009

**Figure 1:**
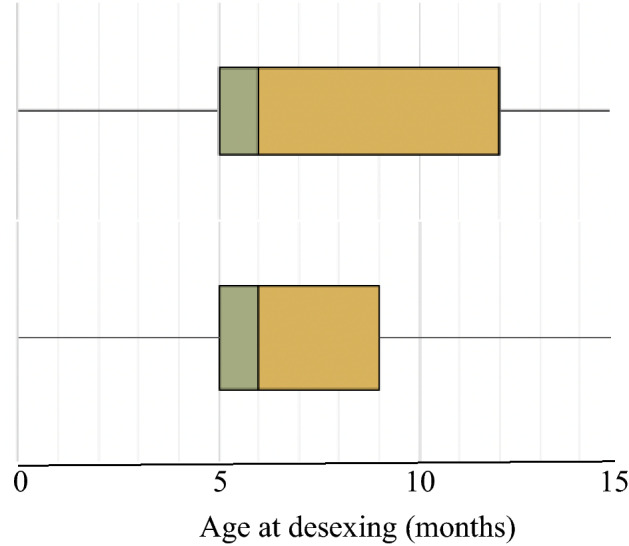
Age at desexing surgery for cats born from 1995 to 2009 (top) and 2010–2017 (bottom). Median and interquartile ranges are shown.

**Figure 2:**
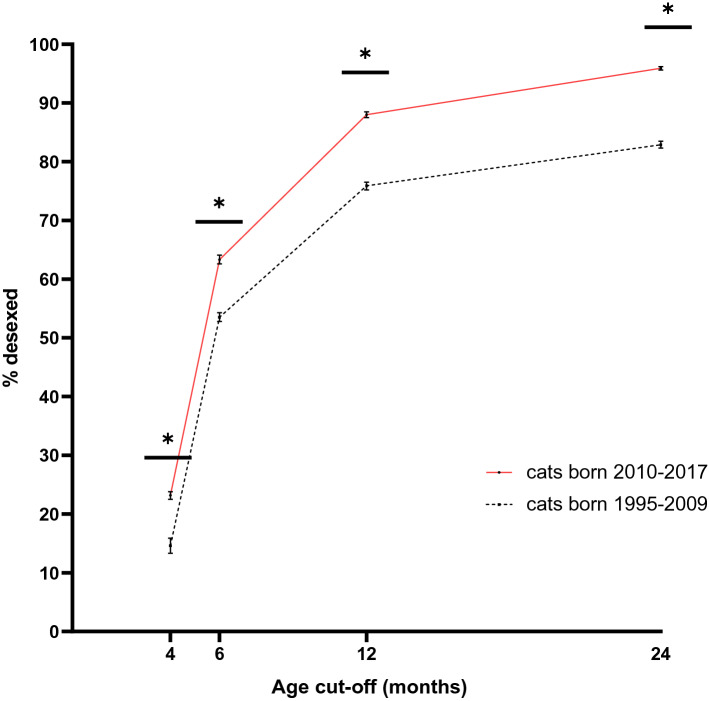
Comparison of the proportion of cats desexed before 4, 6, 12 or 24 months of age in Australian cat cohorts born in consecutive time periods. Vertical bars represent CI_95_. * denotes *p* < 0.05.

Age at desexing was identifiable for 2808 cats born between January 1st 1995 and December 31st 2009 inclusive. Sex and breed, recorded for 99.9% of the population, showed 48.6% males, and a majority of mixed breed cats (77.7%). The median age at which cats were desexed was 6 months old (range < 1 to 238 months) (Fig. 1). Of this desexed cat population, 14.6% had been desexed by 4 months of age, 53.6% by 6 months of age, 75.9% by 1 year and 24.1% after 1 year of age (Fig. 2).

#### Comparison between cohorts

The mean age at desexing in cats born 2010–2017 (8.27 months, ± 0.13[SE]) was significantly lower than the mean age at desexing among those born from 1995 to 2009 (21.49 months, ± 1.41) (*p* < 0.001). In cats born from 2010 to 2017 the odds of being desexed before each age cut-off were significantly greater than for those born from 1995 to 2009; ≤ 4 months OR 1.76 (1.58–1.97) *p* < 0.001, ≤ 6 months OR 1.50 (1.38–1.62), ≤ 1 year OR 2.33 (2.11–2.57) *p* < 0.001.

## Discussion

This is the first large scale analysis of feline desexing practices in Australia using outcomes documented in the patient medical record. The findings complement those of previous studies that used survey data to analyse the attitudes and opinions of veterinary professionals and owners to desexing^31,34,38^. The prevalence of desexing among cats in Australia, found to be 83.6%, confirms that desexing rates in Australia are among the highest reported internationally. Survey-based studies have reported that approximately 90% of cats in Australia are desexed, compared with 80% in the USA, and 43% in Italy^39–45^. A recent EPR-based study conducted in the UK reported the prevalence of feline desexing as 77%^46^. While population-wide analyses of desexing status provide a useful snapshot of practices in a region, most do not consider reproductive history.

A clear shift over time towards desexing cats at a younger age was evident here. EAD was 1.76 times more likely to have been carried out among desexed cats born between 2010 to 2017, than in those born between 1995 and 2009. This move towards earlier desexing was apparent in all age groups studied. Despite this trend, EAD had been carried out in only 21.5% of desexed females in the recent period. In fact, only 59.8% of females had been desexed by 6 months of age, which is the traditional recommendation and the most common recommendation reported by vets in Australia^31,47^. Despite a move towards earlier desexing, opportunities to control reproduction by prepubertal desexing are still being lost.

For an individual female cat, desexing at 6 months or later may be of little consequence, since they may not yet have reached puberty or had access to a mate. A recent survey of cat owners in Australia and New Zealand however found that 66% of cats had outdoor access^45^. From a population control perspective, eliminating the possibility of pregnancy by adopting EAD as standard has merit. The body of scientific evidence generated specifically to address the short-term and long-term safety of EAD overwhelmingly validates this practice^4,17,22–29^.

The impact that tighter control of reproduction among owned cats would have on shelter and stray populations is not yet clear. Populations of owned cats (completely reliant on humans) feral cats (living independently of humans) and stray cats (intermediate relationship with humans) do not exist in isolation^48^. Anthropogenic factors, including the provision of food, abandonment, and failure to curb reproduction, influence cat abundance and movement through these populations. Modelling population dynamics in owned, unowned (stray and feral) and shelter-housed cats holds promise to inform cat management strategies in the future^49^.

In multivariable models, for cats born 2010–2017, sex, breed, state and socioeconomic indices were all significantly associated with both desexing status and age at surgery. Females were less likely than males to be desexed and, among desexed cats, females were less likely than males to have been desexed at ≤ 4 months, supporting future measures to promote EAD in female cats. The reasons for this difference were not investigated but, conceivably, it may be due to higher fees for desexing females at some practices, or a greater awareness of spraying and roaming behaviours in males than pregancies in young female cats.

Not surprisingly purebred cats were less likely to be desexed than mixed breeds. In contrast, the finding that purebred cats were 2.7 times less likely to undergo EAD was unexpected because breeders commonly request EAD so that progeny for the pet market can be sold without delay^50^. It is plausible that this result reflects a greater demand in Australia for EAD from the charity and shelter sector, where mixed breed cats predominate, than from breeders. In line with this possibility, recent surveys found 70–80% of cats in Australia and New Zealand are of mixed breed and acquired from shelters, veterinary clinics, friends and as strays^40,45,51^. The higher odds of EAD in males than females was even greater among purebred cats, a result that may have been influenced by the practice of retaining more entire females than males for breeding.

The breeding season in Australia and New Zealand extends year round with peaks of kittening in spring and summer inferred from shelter admissions^9,52,53^. Cats born in winter had the lowest odds of being desexed in each age group. One explanation for this finding is that promotion of desexing by veterinary practices and welfare groups is less likely in winter because fewer kittens are born. This seasonal difference is certainly seen in the UK, where the RSPCA conducts desexing campaigns in Autumn to prevent the peak of spring litters^54^.

State or territory influenced both whether a cat was desexed, and the odds of EAD. Compared with cats in New South Wales (NSW), those in Victoria (VIC) and South Australia (SA) were more likely, and those in Queensland (QLD) less likely to be desexed. Again, compared with NSW, the odds of being desexed at ≤ 4 months were 1.45 greater for cats in VIC and 1.5–2.3 times less for those in QLD, SA and ACT. Desexing is handled inconsistently between Australia’s states and territories. Mandatory desexing legislation exists in ACT (by 3 months of age) and in SA, Tasmania (TAS), WA (by 6 months of age), with some exceptions. No legal requirement to desex cats exists at state level in VIC, QLD, NSW or Northern Territory (NT), although desexing is indirectly incentivized in NSW (by 6 months of age) and VIC (by 3 months of age) where registration is mandatory, and reduced registration fees are applied for desexed cats. No consistent relationship between our findings and state legislation related to desexing cats was identified. In fact, in ACT, where desexing of pet cats at 3 months of age has been a legal requirement since 2007, the second lowest odds of EAD were identified. Most veterinarians practicing in ACT (90%), surveyed 10 years after the legislation was introduced, gave recommendations inconsistent with the legislation and 35% were unaware that desexing by 3 months was mandatory in the ACT^34^. Whether and how legislation might be an appropriate tool to influence reproduction in owned cats and, indirectly, overpopulation should be further investigated.

Socioeconomic conditions influenced both whether a cat was desexed or not, and the age at desexing. Entire cats were more common in remote, low income and disadvantaged areas. This finding is concerning, given that outdoor access was more likely in non-urban than urban areas in a study of households in Australia and New Zealand^45^, implying more opportunity to find a mate. In addition, stray cat density correlated positively with socioeconomic deprivation in a New Zealand-based study employing geographically weighted regression analyses^55^. Together, these findings support the promotion of desexing campaigns in non-urban areas.

Economic indicators such as household income influenced whether a cat was desexed; the odds of being desexed were around 1.2 times greater in the highest compared to the lowest income areas. A similar, but more dramatic effect was reported in a study conducted in the USA where the prevalence of desexing increased from 51.4% to 96.2% as household income increased^43^. Among desexed cats, EAD was least likely in low income areas, but highest in the most socio-economically disadvantaged areas. Although this might seem paradoxical, IRSD is based on broader indicators of disadvantage than income alone. A UK study, similarly, identified that EAD was most likely in the most deprived regions, and that chances of being desexed by 6 months were more likely in higher income areas^56^. Possible explanations for these observations include the preferential targeting of areas of greatest disadvantage, rather than those with fewer economic resources, by discount desexing programs promoting EAD, or preferential sourcing of kittens in disadvantaged areas from organizations that routinely practice EAD, such as shelters.

There are limitations to our study that should be considered when interpreting the results. Cats that were either not registered with a veterinary practice, or were registered with a practice that did not contribute to VCA during the study periods were not studied. Therefore actual desexing prevalences are almost certainly lower than the estimates reported here. The study population represents cats that are accessible for desexing and is expected to comprise cats kept as pets, for breeding, owned by shelters, semi-owned cats and others. Provenance and lifestyle were not investigated because we chose not to collect data from the examination text field in VCA because of its low positive predictive value^57^, and because these data are inconsistently recorded. This precluded the analysis of other variables that may have been related to desexing outcomes such access to outdoors and the number and species of pets. Data collection was not uniform across Australia and variations in sample size, for example between states, may have affected our results. Also it is possible that data for the same cat presenting at more than one practice could be counted more than once, although a previous study using VCA found that < 0.01% of the data points were affected by this potential source of error^57^.

In conclusion, most cats presented to veterinarians in Australia are desexed. Surgery is being carried out earlier than previously but many cats will have had opportunities to reproduce before they are desexed. Predictors of desexing status and age at surgery identified here will support the effective use of resources when designing targeted strategies to promote EAD, the impact of which can be evaluated by large scale EPR analyses in the future.

## Methods

### Database management

Initial analysis of 1500 randomly-selected records identified variables amenable to analysis in a larger dataset. Data fields included date of birth, breed, sex, desexing status, client postcode, invoice items and consultation date. Age at desexing was derived using invoice data. Data from domestic cats with unique patient identifiers seen at first opinion veterinary clinics across Australia between 2^nd^ January 1993 and 9^th^ November 2018 inclusive, with desexing status recorded, were extracted from the VCA database.

### Study populations

To investigate the prevalence and predictors of desexing, cats born between January 1st 2010 and December 31st 2017 inclusive with desexing status recorded were identified, (Outcome 1; being desexed or not). To investigate the age at which desexing was performed and its predictors, two cohorts of cats were identified (i) desexed cats born between January 1st 2010 and December 31st 2017 inclusive, and (ii) desexed cats born between January 1st 1995 and December 31st 2009 inclusive (Outcome 2: being above or below the age cut-offs; < 4 months, < 6 months, < 1 year < 2 years as binary variables).

### Data retrieval and recoding

Raw data were retrieved using the *describe* function of the *questionr* R package^58^ and data cleaning and recoding performed in Python. For example, sex was recoded as Male, Female or not available (NA) by searching for these terms and variants thereof. Season of birth was derived from the date of birth. In Australia, Spring extends from September to November inclusive; Summer, from December to February; Autumn, from March to May; and Winter, from June to August, as defined by the Australian Bureau of Meteorology^59^. Age was calculated in months and age at desexing was analysed in the following age groups; < 4 months, < 6 months, < 1 year < 2 years and > 2 years. RA, a measure of relative access to services, IER and IRSD were derived from postcode using the Socio-Economic Indexes for Areas tool, Australian Bureau of Statistics^60,61^. RA data were handled as Major Cities of Australia (MCA) and Other. IER and IRSD values 1 to 10 were grouped into quantiles. A low IER score correlates with a high prevalence of households with low income, whereas a higher score indicates relatively greater access to financial resources. A low score for IRSD indicates a high proportion of disadvantaged households, whereas a higher score indicates less disadvantage, but not greater advantage per se. The Python script was run using Pyzo 4.7.3 (binary) on the 3.7.2 version of Python on MacOS.

### Statistical analyses

To compare the associations of each variable on outcome one (being desexed or not) and outcome two (being above or below the age cut-offs; < 4 months, < 6 months, < 1 year < 2 years as binary variables), univariable logistic regression was performed to generate odds ratios (OR) and corresponding 95% confidence intervals (CI_95_). The multivariable model was built by excluding variables based on their effect on the Akaike Information Criterion. The *glmulti* function was then used to identify plausible interactions that could improve the AIC, and the effect of these interactions was checked manually. A multivariable logistic regression was performed on the final model.

To compare the historical data, the mean age at desexing, in months, was obtained for each population. The means were compared using a Welsh’s t-test due to their normal distribution and non-homogenous differences. The proportion of cats desexed in each age cut-off was compared using univariable logistic regression with resultant odds ratios and CI_95_ calculated. Data analysis and statistical comparison were performed using RStudio 1.2.1335 (RStudio Inc.) and Excel for Mac 16.27 (Microsoft). Statistical significance was set at *p* < 0.05.
